# Analysis of the machinery and intermediates of the 5hmC-mediated DNA demethylation pathway in aging on samples from the MARK-AGE Study

**DOI:** 10.18632/aging.101022

**Published:** 2016-08-29

**Authors:** Elisabetta Valentini, Michele Zampieri, Marco Malavolta, Maria Giulia Bacalini, Roberta Calabrese, Tiziana Guastafierro, Anna Reale, Claudio Franceschi, Antti Hervonen, Bernhard Koller, Jürgen Bernhardt, P. Eline Slagboom, Olivier Toussaint, Ewa Sikora, Efstathios S. Gonos, Nicolle Breusing, Tilman Grune, Eugène Jansen, Martijn E.T. Dollé, María Moreno-Villanueva, Thilo Sindlinger, Alexander Bürkle, Fabio Ciccarone, Paola Caiafa

**Affiliations:** ^1^ Department of Cellular Biotechnologies and Hematology, Faculty of Pharmacy and Medicine, Sapienza University of Rome, Rome 00161, Italy; ^2^ Pasteur Institute-Fondazione Cenci Bolognetti, Rome 00161, Italy; ^3^ National Institute of Health and Science on Aging (INRCA), Nutrition and Ageing Centre, Scientific and Technological Research Area, 60100 Ancona, Italy; ^4^ Department of Experimental, Diagnostic and Specialty Medicine, Alma Mater Studiorum-University of Bologna, Bologna 40126, Italy; ^5^ CIG-Interdepartmental Center “L. Galvani”, Alma Mater Studiorum, University of Bologna, 40126 Bologna, Italy; ^6^ The School of Medicine, The University of Tampere, 33014 Tampere, Finland; ^7^ Department for Internal Medicine, University Teaching Hospital Hall in Tirol, Tirol, Austria; ^8^ BioTeSys GmbH, 73728 Esslingen, Germany; ^9^ Department of Molecular Epidemiology, Leiden University Medical Centre, Leiden, The Netherlands; ^10^ University of Namur, Research Unit on Cellular Biology, Namur B-5000, Belgium; ^11^ Laboratory of the Molecular Bases of Ageing, Nencki Institute of Experimental Biology, Polish Academy of Sciences, 02-093 Warsaw, Poland; ^12^ National Hellenic Research Foundation, Institute of Biology, Medicinal Chemistry and Biotechnology, Athens, Greece; ^13^ Institute of Nutritional Medicine (180c), University of Hohenheim, 70599 Stuttgart, Germany; ^14^ German Institute of Human Nutrition Potsdam-Rehbruecke (DIfE), 14558 Nuthetal, Germany; ^15^ Centre for Health Protection, National Institute for Public Health and the Environment, 3720 BA Bilthoven, The Netherlands; ^16^ Molecular Toxicology Group, Department of Biology, University of Konstanz, 78457 Konstanz, Germany; ^17^ Department of Biology, University of Rome “Tor Vergata”, 00133 Rome, Italy

**Keywords:** aging, DNA hydroxymethylation, TET genes, TDG

## Abstract

Gradual changes in the DNA methylation landscape occur throughout aging virtually in all human tissues. A widespread reduction of 5-methylcytosine (5mC), associated with highly reproducible site-specific hypermethylation, characterizes the genome in aging. Therefore, an equilibrium seems to exist between general and directional deregulating events concerning DNA methylation controllers, which may underpin the age-related epigenetic changes. In this context, 5mC-hydroxylases (TET enzymes) are new potential players. In fact, TETs catalyze the stepwise oxidation of 5mC to 5-hydroxymethylcytosine (5hmC), 5-formylcytosine (5fC) and 5-carboxylcytosine (5caC), driving the DNA demethylation process based on thymine DNA glycosylase (TDG)-mediated DNA repair pathway. The present paper reports the expression of DNA hydroxymethylation components, the levels of 5hmC and of its derivatives in peripheral blood mononuclear cells of age-stratified donors recruited in several European countries in the context of the EU Project ‘MARK-AGE’. The results provide evidence for an age-related decline of *TET1, TET3* and *TDG* gene expression along with a decrease of 5hmC and an accumulation of 5caC. These associations were independent of confounding variables, including recruitment center, gender and leukocyte composition. The observed impairment of 5hmC-mediated DNA demethylation pathway in blood cells may lead to aberrant transcriptional programs in the elderly.

## INTRODUCTION

DNA methylation refers to the conversion of cytosine to 5-methylcytosine (5mC) by the activity of the DNA methyltransferase enzymes (namely DNMT1, DNMT3A and DNMT3B). The preservation of the epigenetic code introduced by DNA methylation is not only necessary for shaping proper transcriptional networks of each specific cell type but also for assuring genomic stability [1]. During development, cellular differentiation and senescence, profound changes of DNA methylation patterns occur, allowing the repression or activation of specific genes through hypermethylation or demethylation of their promoters and/or distal regulatory sequences [2,3]. Chronological alteration of DNA methylation patterns is a well-recognized hallmark of aging, identifiable in most of human tissues [4]. Notably, similar epigenetic alterations are typical of pathological conditions highly associated with age, including cancer and neuro-degeneration [5,6]. The alterations of DNA methylation in aging are characterized by the co-occurrence of DNA demethylation and hypermethylation events. The genome undergoes a global loss of methylation together with the acquisition of site-specific hypermethylation, which prevalently concerns the CpG islands (CGI) [6–8]. Notably, the presence of specific hot-spots of age-associated differential methylation led to the possibility of exploiting DNA methylation as a biomarker of biological aging [4,8,9].

The transcriptional deregulation of DNMT enzymes has been proposed as one of the potential causes of the aging-associated changes of DNA methylation patterns. Impairment of DNMT transcription and/or activity could result from interaction with environmental factors, including life-style choices and dietary habits [8,10].

The recent discovery of 5-hydroxymethylcytosine (5hmC) has, however, increased the complexity of epigenetic events influencing DNA methylation patterns. 5hmC derives from the oxidation of 5mC by the action of the Fe2^+^- and 2-oxoglutarate-dependent dioxygenases TET1, TET2 and TET3 [11,12]. TET enzymes show distinct and overlapping functions in terms of stage- and organ-specific functions [11,12] as well as in patterning the distribution of 5mC and 5hmC signals in the genome [13]. Global levels of 5hmC are highly variable in human tissues. Brain represents the tissue with the highest content of 5hmC [11,14]. Compelling evidence indicates that 5hmC has a dual role on the genome, acting both as an epigenetic mark and an intermediate of DNA demethylation. Consistently, 5hmC is now considered as “the sixth base” of genome (with 5mC being “the fifth base”) playing important functions in the regulation of transcription. On the other hand, 5hmC is also pivotal for starting active DNA demethylation processes [11,12]. In fact, all TET enzymes can catalyze the sequential oxidations of 5mC initially into 5hmC and then into 5-formylcytosine (5fC) and 5-carboxyl-cytosine (5caC) [15], thus triggering the substitution of 5mC derivatives with unmethylated cytosines by DNA repair mechanisms. The base excision repair (BER) pathway, through the action of the thymine DNA glycosylase (TDG), seems to be largely involved in the removal of 5fC and 5caC from DNA [16]. In addition, the TET-mediated oxidations of 5mC may also lead to passive DNA demethylation, as DNMTs fail to recognize 5hmC, 5fC or 5caC during the replication-associated process of DNA methylation maintenance [17,18]. Deregulation of TET enzymes, followed up by either DNA repair or DNA replication, may contribute to the anomalous DNA methylation patterns observed in aging via the 5hmC intermediate. However, efforts addressed to this topic are still very limited and focused on brain tissues, where an increase of 5hmC levels has been mainly observed with age [19,20].

In the present work, the association of 5hmC with aging has been investigated in peripheral blood mononuclear cells (PBMC), an easily accessible biological sample whose DNA methylation dynamics with age have been largely characterized [7]. In particular, the expression of members of the DNA demethylation machinery, including TET enzymes and TDG, as well as the levels of 5hmC and its derivatives have been evaluated in individuals aged 34-74 years enrolled for the MARK-AGE Project, an Europe-wide cross-sectional population study aimed at the identification of biomarkers of aging [21,22].

## RESULTS

### Study population

The sample of study consisted of PBMC from 188 volunteers enrolled in eight European countries (i.e. Austria, Belgium, Finland, Germany, Greece, Italy, The Netherlands, Poland) covering the age range between 34 and 74 years. The population was stratified into three age groups (i.e. 34-48; 49-65; 66-74) sharing comparable sample size and gender composition. However, female representation was slightly larger than males in the 34-48 age class. In all age classes the mean value of the body mass index (BMI) was largely below 30 kg/m^2^, which is the threshold for obesity. Moreover, in agreement with literature data, the BMI appeared to increase with age indicating that the population analyzed was representative of a physiological aging process. Another feature given in Table 1 includes self-reported smoking status of the population studied, which was almost similar between age groups. About the half of the subjects had never smoked while the remaining part consisted of current or former smokers (Table 1).

**Table 1:** Characteristics of the study population by age groups^1^

		ALL	Age groups		
	N	188	67	60	61
**Age** (y)	Range	**34-74**	**34-48**	**49-65**	**66-74**
	Mean	55.6	40.4	57.5	70.5
**Gender** % (N)	Male	47 (88)	42 (28)	47 (28)	52 (32)
**BMI** (kg/m^2^) % (N)	Mean ± SD	25.8 ± 4.5	24.9 ± 4.2	25.8 ± 4.5	26.7 ± 4.7
	< 25	50 (93)	60 (40)	53 (32)	35 (21)
	25 to 30	35 (66)	24 (16)	30 (18)	52 (32)
	≥ 30	15 (29)	16 (11)	17 (10)	13 (8)
**Smoking Habits** % (N)	never	52 (97)	58 (39)	46 (28)	49 (30)
	former	33 (63)	21 (14)	37 (22)	44 (27)
	current	15 (28)	21 (14)	17 (10)	7 (4)
**Country** % (N)	Austria	14 (26)	13 (9)	15 (9)	13 (8)
	Belgium	10 (19)	1 (1)	7 (4)	23 (14)
	Italy	24 (45)	40 (27)	18 (11)	11 (7)
	Finland	5 (9)	1 (1)	5 (3)	8 (5)
	Germany	16 (31)	12 (8)	27 (16)	11 (7)
	Greece	16 (29)	22 (15)	15 (9)	8 (5)
	The Netherlands	6 (12)	0 (0)	3 (2)	16 (10)
	Poland	9 (17)	9 (6)	10 (6)	8 (5)

1 Values are mean ± SD and percentage (number). (y)=years.

### Characterization of data values

The parameters analyzed were tested for normal distribution and most of them failed to pass the Kolmogorov-Smirnov and Shapiro-Wilk normality tests (data not shown). Due to skewness and high kurtosis of non-transformed values, data were log-transformed but, also in this case, parameters did not show normal distribution. As such, both parametric and non-parametric tests were applied to verify the robustness of correlations and the results of group comparisons. The generalized linear model (GLM) analysis was performed to investigate the influence of covariates and multiple confounding factors, including recruitment center, gender and the lymphocyte to monocyte (lympho/mono) ratio. The latter was taken into account as PBMC are a mixed cell population, mainly characterized by lymphocytes and monocytes, and the relative composition of each cell type has been demonstrated to affect methylation studies [10,23]. Furthermore, to rule out the possibility of misleading interpretation of data due to potential batch effects, the Partek Genomic Suite 6.6 ANOVA tool was run on log-transformed values adopting two diverse correction procedures: (1) by using default settings; (2) by attempting to retain age and gender differences [10].

### Age-dependent expression of *TET* and *TDG* genes in PBMC

**Figure 1:**
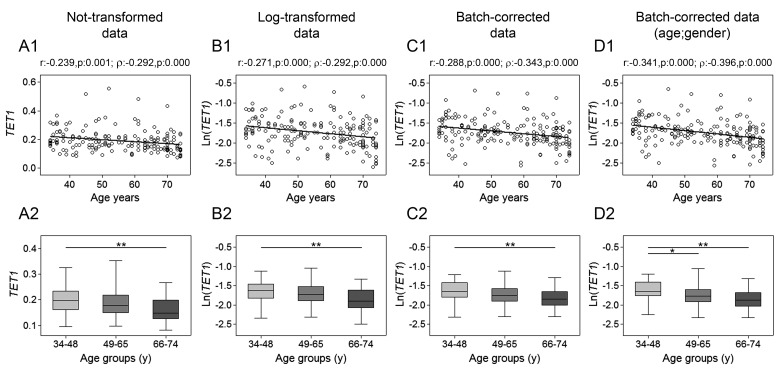
Age-related changes of *TET1* mRNA levels in PBMC Upper panels show scatter plots representing the linear correlation between *TET1* mRNA levels and age in PBMC calculated from (**A1**) non-transformed *TET1* data, (**B1**) log-transformed *TET1* data, (**C1**) batch-corrected *TET1* data, (**D1**) batch-corrected *TET1* data retaining age and gender differences. Parametric (Pearson r) and non-parametric (Spearman's *ρ*) correlation coefficients and statistical significance are given above each graph. Lower panels show bar graphs reporting the expression levels of *TET1* gene in three different age classes calculated from (**A2**) non-transformed *TET1* data, (**B2**) log-transformed *TET1* data, (**C2**) batch-corrected *TET1* data, (**D2**) batch-corrected *TET1* data retaining age and gender differences. Boxplots show the median, the interquartile range (boxes) and the 5–95% data range (whisker caps). Comparisons between groups were performed by the Kruskal-Wallis test followed by post-hoc Bonferroni test (*P < 0.05; **P < 0.01). (y)= years.

**Figure 2:**
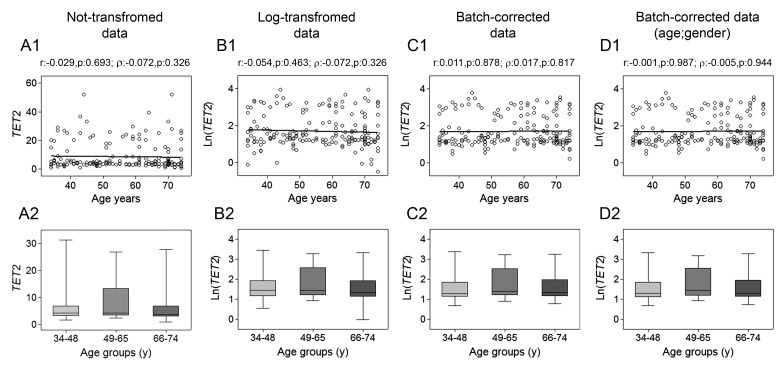
Age-related changes of *TET2* mRNA levels in PBMC Upper panels show scatter plots representing the linear correlation between *TET2* mRNA levels and age in PBMC calculated from (**A1**) non-transformed *TET2* data, (**B1**) log-transformed *TET2* data, (**C1**) batch-corrected *TET2* data, (**D1**) batch-corrected *TET2* data retaining age and gender differences. Parametric (Pearson r) and non-parametric (Spearman's *ρ*) correlation coefficients and statistical significance are given above each graph. Lower panels show bar graphs reporting the expression levels of *TET2* gene in three different age classes calculated from (**A2**) non-transformed *TET2* data, (**B2**) log-transformed *TET2* data, (**C2**) batch-corrected *TET2* data, (**D2**) batch-corrected *TET2* data retaining age and gender differences. Boxplots show the median, the interquartile range (boxes) and the 5–95% data range (whisker caps). Comparisons between groups were performed by the Kruskal-Wallis test followed by post-hoc Bonferroni test. (y)= years.

**Figure 3:**
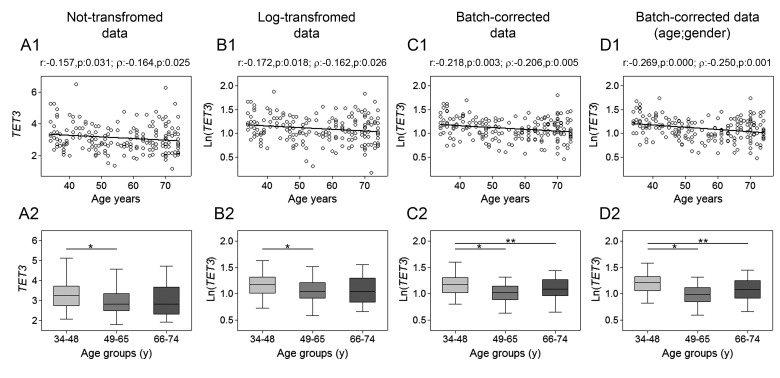
Age-related changes of *TET3* mRNA levels in PBMC Upper panels show scatter plots representing the linear correlation between *TET3* mRNA levels and age in PBMC calculated from (**A1**) non-transformed *TET3* data, (**B1**) log-transformed *TET3* data, (**C1**) batch-corrected *TET3* data, (**D1**) batch-corrected *TET3* data retaining age and gender differences. Parametric (Pearson r) and non-parametric (Spearman's *ρ*) correlation coefficients and statistical significance are given above each graph. Lower panels show bar graphs reporting the expression levels of *TET3* gene in three different age classes calculated from (**A2**) non-transformed *TET3* data, (**B2**) log-transformed *TET3* data, (**C2**) batch-corrected *TET3* data, (**D2**) batch-corrected *TET3* data retaining age and gender differences. Boxplots show the median, the interquartile range (boxes) and the 5–95% data range (whisker caps). Comparisons between groups were performed by the Kruskal-Wallis test followed by post-hoc Bonferroni test (*P < 0.05; **P < 0.01). (y)= years.

The assessment of *TET1*, *TET2*, *TET3* and *TDG* genes mRNA expression was performed by RT-qPCR. Spearman's correlation analysis yielded a highly significant negative linear association between *TET1* expression and age for both non-transformed and log-transformed values, which was even more pronounced after batch effect correction. Pearson's correlation analysis also supported a significant linear decline of *TET1* transcript with age (Fig. 1, upper panels). No association with age was observed for *TET2* expression (Fig. 2), whose values showed high variance. According-ly, cluster analysis performed on *TET2* data identified two subgroups separated around the 75th percentile value (Supplementary Fig.1A and B), where the one with higher *TET2* levels showed a median value six times greater than the other subgroup (data not shown). These two subgroups of *TET2* expression data were correlated separately to age but, even in this case, no association was obtained (Supplementary Fig.1C and D). Regarding *TET3*, both parametric and non-parametric analyses revealed a negative correlation with age, which was even more evident after the removal of batch effects (Fig. 3, upper panels). The relationship of *TET1* and *TET3* genes with age was further tested against gender and center by bootstrapped regression analysis (Supplementary Table 1 and 2). Notably, the negative correlation of *TET1* and *TET3* with age was retained in all conditions, indicating that even if an influence of gender and geographical origin could exist, age affects their expression almost independently. The same analyses confirmed the lack of association with age of both *TET2* and *TET2* expression clusters independently of gender and recruitment center (data not shown).

Additional evidence of a link between aging and *TETs* gene expression was obtained by stratifying samples into three age classes. The age group including the younger individuals (34-48y) showed significantly higher expression of both *TET1* and *TET3* compared to the 66-74y and the 49-65y age groups, respectively.

This was even more evident after correction of batch effects when preserving gender and age differences (Fig. 1 and 3, lower panels). Thereafter, more detailed analyses were adopted to determine the influence of covariates and multiple confounding factors including recruitment center, gender and lympho/mono ratio on the age group differences. In particular, GLM analysis revealed that the *TET1* expression difference between age groups resisted the confounding factors in the case of batch effect corrections (Supplementary Table 3). Due to the notable impact of leukocyte composition of PBMC on *TET1* expression, bootstrapping-coupled regression analysis was performed for *TET1* and age stratifying for the lympho/mono ratio. This analysis definitively demonstrated that age independently affected *TET1* expression (Supplementary Table 4). Similar approaches confirmed that the expression of *TET2* was not associated with age but rather affected by lympho/mono ratio (Fig. 2, lower panels, and Supplementary Table 5). Regarding *TET3* expression, it was sensitive to all tested variables, especially to gender, but none of them affected the observed differences between age groups (Supplementary Table 6).

**Figure 4:**
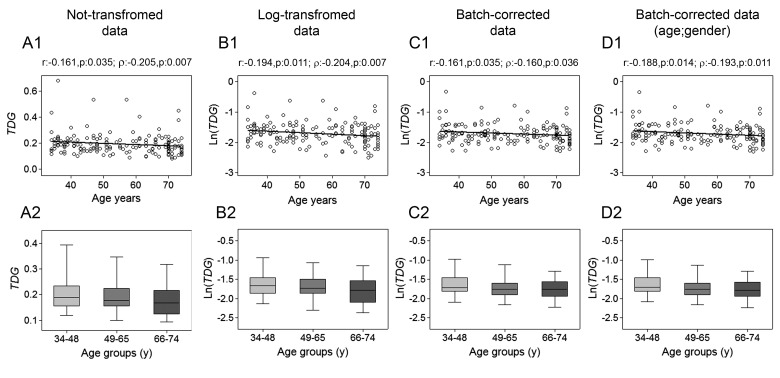
Age-related changes of *TDG* mRNA levels in PBMC Upper panels show scatter plots representing the linear correlation of between *TDG* mRNA levels and age in PBMC calculated from (**A1**) non-transformed *TDG* data, (**B1**) log-transformed *TDG* data, (**C1**) batch-corrected *TDG* data, (**D1**) batch-corrected *TDG* data retaining age and gender differences. Parametric (Pearson r) and non-parametric (Spearman's *ρ*) correlation coefficients and statistical significance are given above each graph. Lower panels show bar graphs reporting the expression levels of *TDG* gene in three different age classes calculated from (**A2**) non-transformed *TDG* data, (**B2**) log-transformed *TDG* data, (**C2**) batch-corrected *TDG* data, (**D2**) batch-corrected *TDG* data retaining age and gender differences. Boxplots show the median, the interquartile range (boxes) and the 5–95% data range (whisker caps). Comparisons between groups were performed by the Kruskal-Wallis test followed by post-hoc Bonferroni test. (y)= years.

The analysis of *TDG* expression revealed a slight negative correlation with age (Fig. 4, upper panels), which was also maintained after batch effect corrections. Regression analysis with bootstrapping supported the decline of *TDG* with increasing age (Supplementary Table 7), which probably was too moderate for being endorsed by the analysis by age classes (Fig. 4, lower panels). GLM analysis uncovered an influence of the recruitment center on *TDG* expression (Supplementary Table 8).

### DNA methylation analysis of *TET1* and *TDG* genes

**Figure 5:**
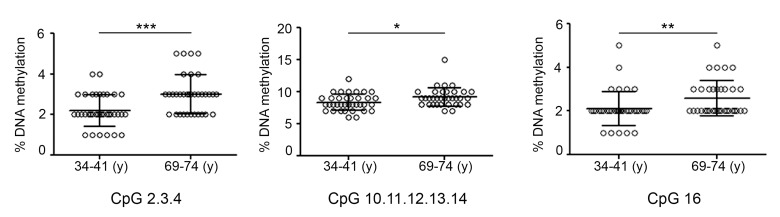
DNA methylation profile of *TET1* CGI in aging Graphs represent the CpGs of *TET1* CGI analyzed by the epiTYPER assay and show the difference in DNA methylation between the groups of young (34-41) and old (69-74) individuals. Statistical significance was obtained by the Mann-Whitney test (*P < 0.05; **P < 0.01; ***P < 0.001). n(34-41y)=36; n(69-74y)=34. (y)= years.

Focal DNA hypermethylation events are part of age-associated changes of DNA methylation patterns and, if occurring on transcriptional regulatory regions, they may lead to down-regulation of gene expression [8]. DNA methylation-mediated control of the genes showing age-related decline has only been demonstrated for *TET1* and *TDG*. In particular, hypermethylation of *TET1* and *TDG* CGIs but also of the *TET1* CGI 3′-shore has been associated with transcriptional repression [24–27]. Based on this evidence, MassARRAY EpiTYPER platform was used to determine the DNA methylation profile of *TET1* and *TDG* genes in young (34-41y) and old (69-74y) individuals. The analysis of the *TET1* CGI revealed a slight but significant hypermethylation of some CpGs in the elderly group (Fig. 5). On the contrary, no other CpGs, neither in the *TET1* CGI 3′-shore nor in the *TDG* CGI, showed any statistically significant change of the DNA methylation levels (Supplementary Fig. 2).

### Content of 5hmC and its derivatives in PBMC in aging

**Figure 6:**
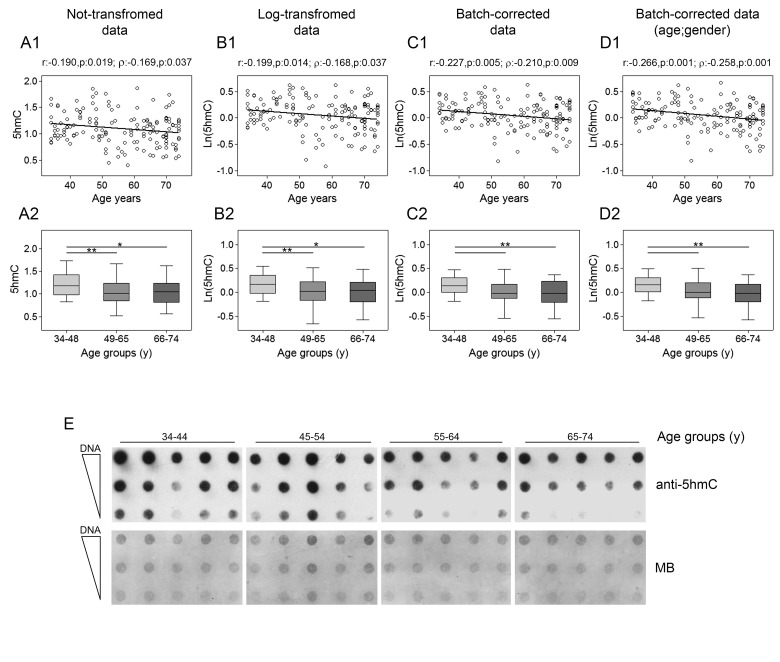
Age-related changes of 5hmC levels in PBMC Upper panels show scatter plots representing the linear correlation of between 5hmc levels and age in PBMC calculated from (**A1**) non-transformed 5hmC data, (**B1**) log-transformed 5hmC data, (**C1**) batch-corrected 5hmC data, (**D1**) batch-corrected 5hmC data retaining age and gender differences. Parametric (Pearson r) and non-parametric (Spearman's *ρ*) correlation coefficients and statistical significance are given above each graph. Lower panels show bar graphs reporting the levels of 5hmC in three different age classes calculated from (**A2**) non-transformed 5HMC data, (**B2**) log-transformed 5hmC data, (**C2**) batch-corrected 5hmC data, (**D2**) batch-corrected 5hmC data retaining age and gender differences. Boxplots show the median, the interquartile range (boxes) and the 5–95% data range (whisker caps). Comparisons between groups were performed by the Kruskal-Wallis test followed by the post-hoc Bonferroni test (*P < 0.05; **P < 0.01). (**E**) Representative dot blot performed on DNA from 20 individuals by using anti-5hmC antibody and methylene blue (MB) staining to control DNA loading. (y)= years.

Analysis of global content of 5hmC, performed by dot blot assay with specific antibodies to 5hmC, showed that its levels decreased linearly with age as demonstrated by Pearson's and Spearman's correlations for both non-transformed and log-transformed data. This result was even more evident after correction of batch effects (Fig. 6, upper panels). Regression analysis performed by using bootstrap resampling stratified for gender and recruitment center supported the solid negative association between age and 5hmC content (Supplementary Table 9). Confirmatory results were also obtained when samples were stratified in age classes. In fact, the group of younger individuals (34-48y) was significantly different from the remaining two groups (49-65y and 66-74y) (Fig. 6, lower panels). GLM analysis confirmed the negative association of 5hmC with age and also revealed a potential impact of recruitment center with no consequences on the age-dependent reduction of 5hmC though (Supplementary Table 10).

**Figure 7:**
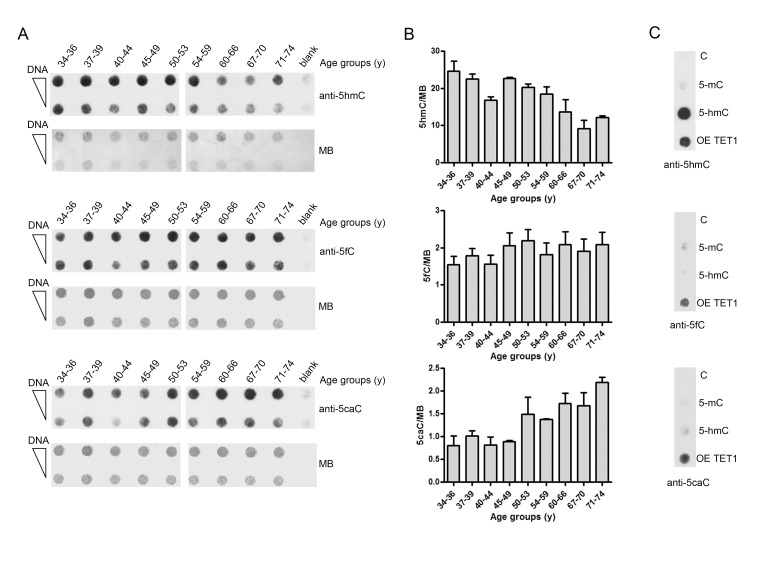
Age-related changes of 5hmC, 5fC and 5caC levels in PBMC (**A**) The graph shows the amount of 5hmC, 5fC and 5caC determined by dot-blot assay on pooled DNA samples obtained by grouping individuals into nine different age classes. n(34-36y)=16; n(37-39y)=17 ; n(40-44y)=16; n(45-49y)=22; n(50-53y)=16; n(54-59y)=16; n(60-66y)=31; n(67-70y)=27; n(71-74y)=27. Analysis was performed with 5hmC, 5fC and 5caC specific antibodies. Methylene blue (MB) staining was used to monitor DNA loading. (**B**) Bar graphs show the densitometric quantification of 5hmC, 5fC and 5caC signal after normalization for loading by MB staining, shown as mean ± S.E.M. of three different technical replicates. (**C**) Linear dsDNA containing unmodified (**C**), full methylated (5mC) or hydroxymethylated (5hmC) cytosines were used as specificity control of the anti-5hmC antibody. DNA derived from HEK293T overexpressing TET1 catalytic domain (OE TET1) was used as positive control for anti-5fC and anti-5caC antibodies. (y)= years.

As TET enzymes are also responsible of the conversion of 5hmC into 5fC and then 5caC, which are considered DNA demethylation intermediates, their levels were also determined by dot-blot analysis. Due to the low genomic content of 5fC and 5caC and the limited amount of DNA available, DNA from multiple samples was pooled into 9 groups of increasing age. This analysis confirmed the decreasing trend of 5hmC with aging and revealed an accumulation of 5caC in older ages. By contrast, the levels of 5fC appeared to be comparable between groups (Fig. 7). Notably, correlation analyses showed a significant negative association between 5hmC and 5caC levels (Pearson r: −0.771; p: 0.015; Spearman *ρ*: −0.767; p: 0.021).

### Correlation between components of DNA demethylation machinery, 5hmC levels and other epigenetic enzymes

A putative correlation between the expression of *TET1-3* and *TDG* and the levels of 5hmC was investigated. A slight significant positive correlation was only detected by non-parametric test between *TET1* expression and 5hmC levels after batch effect correction (Table 2). Further correlation analyses were then performed to trace back a possible co-regulative network involving *TET* genes, *TDG* and other epigenetic factors functionally related to DNA methylation/demethylation pathways. In particular, data from the same subset of PBMC samples were available for DNA methyltransferases *DNMT1* and *DNMT3B* and for poly(ADP-ribose) polymerases *PARP1* and *PARP2*. The most evident associations were obtained for *TET1*, which exhibited significant positive correlations with *TDG*, *DNMT1*, *DNMT3B*, along with *PARP1* and *PARP2*. A strong correlation was also found between *TDG* and *PARP2*. Notably, correlations between these variables were significant after both parametric and non-parametric tests, with or without batch effect corrections. On the contrary, other associations, such as the one between *TET3* and *TET2* or *PARP1*, or between this latter and *TDG*, were not confirmed by either test or they only emerged after the removal of batch effects (Table 2 and 3).

**Table 2:** Correlation between 5hmC levels and components of the DNA demethylation machinery

Pearson p-value	5hmC	*TET1*	*TET2*	*TET3*	*TDG*	5hmC	*TET1*	*TET2*	*TET3*	*TDG*	5hmC	*TET1*	*TET2*	*TET3*	*TDG*
Spearman p-value
**5hmC**		−0.025 0.761	0.098 0.230	−0.049 0.547	−0.088 0.303		−0.018 0.825	0.043 0.597	0.015 0.855	−0.153 0.073		0.049 0.549	0.066 0.419	0.044 0.592	−0.120 0.160
***TET1***	0.043 0.601		0.079 0.284	0.012 0.875	**0.379** 0.000 ***	0.092 0.260		0.040 0.590	0.043 0.562	**0.311** 0.000 ***	**0.159** 0.050 *		0.044 0.548	0.053 0.473	**0.315** 0.000 ***
***TET2***	0.103 0.206	0.138 0.060		0.038 0.608	−0.005 0.947	0.058 0.480	0.102 0.165		0.121 0.098	−0.022 0.779	0.093 0.255	0.106 0.146		0.118 0.106	−0.22 0.779
***TET3***	−0.043 0.594	0.026 0.728	0.126 0.085		**0.158** 0.038 *	−0.027 0.741	0.079 0.278	**0.225** 0.002 **		0.131 0.086	0.022 0.784	0.088 0.231	**0.188** 0.010 **		0.139 0.070
***TDG***	−0.039 0.646	**0.365** 0.000 ***	0.083 0.277	0.101 0.186		−0.058 0.496	**0.266** 0.000 ***	0.027 0.724	0.095 0.217		−0.025 0.770	**0.283** 0.000 ***	0.040 0.606	0.102 0.185	
	Log-transformed data					Log-transformed data (batch corr.)					Log-transformed (batch corr_age;gender)				

The linear association between parameters was calculated using parametric (Pearson r) and non-parametric (Spearman's *ρ*) correlation coefficients considering Log-transformed data; Log-transformed data after batch effect correction (batch corr.); and Log-transformed data after batch effect correction retaining age and gender differences (batch corr_age;gender). Statistical significance:

* p < 0.05

*** p < 0.01

** p < 0.001.

**Table 3:** Correlation between components of the DNA demethylation machinery and other epigenetic enzymes

Pearson p-value	*TET1*	*TET2*	*TET3*	*TDG*	*TET1*	*TET2*	*TET3*	*TDG*	*TET1*	*TET2*	*TET3*	*TDG*
Spearman p-value
***DNMT1***	**0.188** 0.011 * ** 0.215 **0.004**	**0.149** 0.046 * * 0.171 **0.022**	0.013 0.862 0.013 0.867	0.051 0.516 0.064 0.417	**0.387** 0.000 *** *** **0.329** **0.000**	0.023 0.754 0.039 0.600	−0.006 0.938 -0,063 0.404	0.046 0.560 0.102 0.193	**0.405** 0.000 *** *** **0.342** **0.000**	0.033 0.658 0.059 0.428	−0.015 0.837 -0.078 0.299	0.051 0.516 0.097 0.215
***DNMT3B***	**0.285** 0.000 ** *** **0.196** **0.009**	0.012 0.874 0.064 0.393	**0.154** 0.039 * 0.146 0.051	0.111 0.157 0.102 0.193	**0.344** 0.000 *** *** **0.284** **0.000**	−0023 0-764 -0.020 0.794	0.001 0.990 0.003 0.970	0.054 0.491 0.072 0.358	**0.353** 0.000 *** *** **0.305** **0.000**	−0.023 0.757 -0.004 0.960	0.010 0.889 0.007 0.924	0.064 0.413 0.074 0.343
***PARP1***	**0.165** 0.024 *** **0.193** **0.008**	**0.146** 0.046** 0.156 **0.033**	0.030 0.679 -0.045 0.539	0.084 0.273 0.069 0.372	**0.324** 0.000 *** *** **0.282** **0.000**	−0.061 0.418 -0.071 0.347	−0.058 0.436 * **-0.164** **0.028**	**0.161** 0.039 * 0.115 0.144	**0.341** 0.000 *** *** **0.296** **0.000**	−0.052 0.488 -0.043 0.567	−0.043 0.568 * **-0.163** **0.029**	**0.185** 0.018* 0.120 0.126
***PARP2***	**0.358** 0.000 *** *** **0.301** **0.000**	0.058 0.434 0.095 0.196	0.035 0.635 0.028 0.701	**0.440** 0.000 *** *** **0.415** **0.000**	**0.337** 0.000 *** *** **0.276** **0.000**	−0.001 0.988 0.119 0.112	−0.087 0.245 -0.080 0.284	**0.365** 0.000 *** *** **0.303** **0.000**	**0.353** 0.000 *** *** **0.292** **0.000**	−0.002 0.981 0.123 0.101	−0.098 0.193 -0.096 0.200	**0.366** 0.000 *** *** **0.299** **0.000**
	Log-transformed				Log-transformed (batch corr.)				Log-transformed (batch corr_age;gender)			

The linear association between parameters was calculated using parametric (Pearson r) and non-parametric (Spearman's ρ) correlation coefficients considering Log-transformed data; Log-transformed data after batch effect correction (batch corr.); and Log-transformed data after batch effect correction retaining age and gender differences (batch corr age;gender). Statistical significance:

* p < 0.05

*** p < 0.01

** p < 0.001.

### Analysis of clinical variables associated with the bimodal distribution of *TET2* mRNA levels

Apart from data on *TET2* gene expression, all other parameters investigated displayed a homogenous distribution of values and a similar variance. In fact, *TET2* values split the samples into two main subgroups, whose classification in high and low *TET2* expression classes was not ascribable to demographic characteris-tics including gender, age or the country of origin (Supplementary Fig. 1 and Supplementary Table 5).

**Figure 8:**
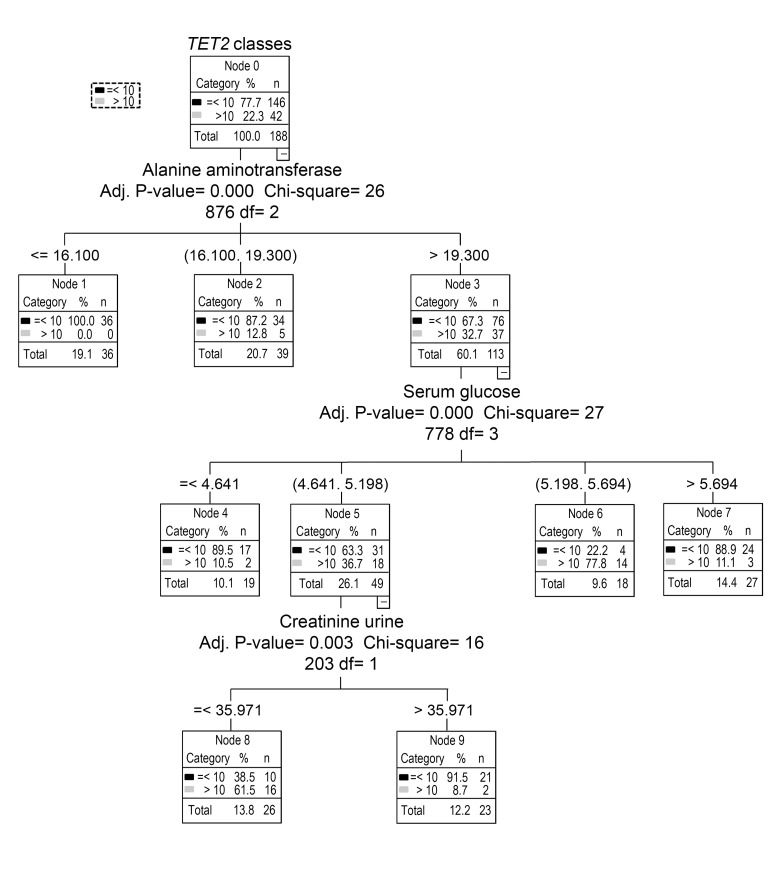
Identification of major variables that affect *TET2* expression by decision tree analysis Decision tree analysis was performed to identify potential variables responsible of *TET2* gene bimodal distribution. Apart from demographic characteristics, several clinical biochemistry parameters were included.

The availability of a large set of clinical parameters for the individuals enrolled prompted the investigation of the possible sources underlying the high variability of *TET2* expression. For this purpose, a decision tree analysis was performed, which identified the serum levels of alanine aminotransferase (ALT) as the most important factor influencing *TET2*. Minor subgroups were determined on the basis of serum glucose and urine creatinine concen-trations. In particular, individuals showing high *TET2* expression also had elevated ALT and intermediate glycaemia while about one third of them showed lower levels of urine creatinine concentrations (Fig. 8). How-ever, as the subdivision of *TET2* values based on serum glucose levels fell within the normoglycemic range and the low creatinine subgroup concerned very few samples, only the relationship of *TET2* with ALT levels was examined further. In particular, GLM analysis, with gender, age, lympho/mono ratio and recruitment center as covariates, definitively confirmed the positive association between ALT levels and *TET2* expression (Table 4).

**Table 4:** Influence of *TET2* classes and other covariates on serum alanine aminotransferase levels†

Test of Model Effects	Alanine aminotransferase		
Variables	Type III		
	Wald Chi-Square	df	Sig.
**(Intercept)**	142.9180	1	< 0.001
**Center**	16.747	7	0.019
**Gender**	26.074	1	< 0.001
**Age groups**	2.238	2	0.327
**Lympho/mono**	3.356	1	0.067
***TET2* class**	10.778	1	0.001

† Analysis was performed by GLM using linear model with identity link-function considering as dependent variable: Alanine amino-transferase. Model: (Intercept), center, gender, age groups, lympho/mono (used as continuous variable); *TET2* classes.

## DISCUSSION

Although the discovery of 5hmC is quite recent compared to that of other epigenetic modifications, relevant achievements have been rapidly reached about its function and that of the TET family enzymes in development, stem cell biology and disease as well [11,12]. In fact, the interest in “the sixth base” of DNA is continuously growing as the impaired formation of 5hmC occurs in several pathologies such as cancer and neurodegenerative disorders. In most cases, changes of 5hmC levels have been ascribed to genetic or transcriptional faults concerning *TET* genes. In cancer, the global loss of 5hmC has been frequently associated with *TET2* somatic mutations in leukemia [28,29] or with *TET1* silencing in solid tumors [25,26]. As far as neurodegenerative disorders are concerned, loss of 5hmC has been described in the striatum and cortex of Huntington's disease brains [30] and in PBMC from multiple sclerosis patients [31]. In contrast, mainly accumulation of 5hmC seems to characterize cerebral cortex and hippocampus of Alzheimer's disease patients [5,32]. It is noteworthy that the hippocampus in aging also shows increasing levels of 5hmC [19,20] and that *TET2* mutations have been found associated with older age [28,29]. In addition, together with Alzheimer's disease and cancer, other age-associated pathological states, including diabetes [33] and atherosclerosis [34] are characterized by deregulated 5hmC levels. Therefore, a partial contribution of age-associated epigenetic changes to the onset of those pathologies is conceivable.

In the present work, the age-associated expression in blood cells of pivotal enzymes involved in active DNA demethylation process has been broadly tested in subjects recruited from the general population of eight European countries. The data indicated that *TET1* and *TET3* gene expression in PBMC undergo a linear decrease with aging whereas *TET2* was not influenced. Evidence of TET enzymes down-regulation in aging has been previously reported in human epidermis for *TET1* [35], in mouse liver for *Tet3* [36] and in human T-cells for both genes [37]. All together these observations indicate that altered level of 5mC-hydroxylases could be a common feature of aging in multiple tissues.

The investigation of demographic variables that could potentially affect the expression of *TET* genes in PBMC indicated that *TET3* expression was sensitive to gender and country of origin. By contrast, expression of *TET1* was influenced by cell type composition of PBMC samples. In particular, high expression of *TET1* was associated with high lympho/mono ratio (data not shown), which is in agreement with the low *TET1* levels observed in human monocytes and monocyte-derived dendritic cells [38]. The effect of distinct leukocyte populations in epigenetic studies based on blood sample analyses have also been reported for DNA methylation patterns [23] and for the expression of the maintenance methyltransferase *DNMT1* as well [10]. Nevertheless, in the case of *TET1* and *TET3*, age basically acts as an independent variable with respect to the effect of other confounding factors.

Hypermethylation of the promoter-associated CGI and of the 3′-shore of *TET1* gene is responsible of its silencing in cancer [24–26]. Moreover, aberrant methylation of the *TET1* CGI 3′-shore was also observed in persons affected by Down Syndrome, a disease with several characteristics of accelerated aging, but no information about the transcriptional outcome of this event is available yet [39]. The possible physio-logical changes in the DNA methylation profile of *TET1* gene regulatory regions was therefore examined in aging.

The results showed hypermethylation of few CpGs within *TET1* CGI in the elderly group. The low differential methylation level observed between young and old people groups does not permit to conclude that the age-dependent decrease of *TET1* in PBMC is caused by the hypermethylation of this region, but it raises the possibility that such epigenetic change pertains to a specific subpopulation of PBMC.

Even though *TET2* expression did not show any particular trend with age, this parameter was interesting as more than a fifth of all samples had remarkable high expression levels. However, this peculiar feature of *TET2* gene could not be traced back to gender and/or recruitment center. On the other hand, the examination of several variables, mainly including clinical laboratory parameters, revealed a potential influence of elevated levels of serum ALT. Notably, the values of ALT showing association with high *TET2* levels were mainly below the upper limit for conventional “normal” ALT range but quite over the one of the updated “healthy” ALT range calculated on individuals having other clinical parameters within reference range [40].

Increased ALT levels are generally associated to hepatic injury and can depend on various causes including hepatitis virus infections, hepatic steatosis but also medications, thyroid disorders and celiac disease [41]. The origin of elevated ALT levels was thus difficult to be determined in the examined population based on the available clinical data of donors. Nevertheless, the interesting *TET2* up-regulation in PBMC, associated with ALT levels, could indicate the need of TET2 immunological functions [42] for orchestrating a specific immune response to liver insults.

The transcriptional analyses revealed that also the expression of *TDG*, which initiates the removal of 5fC or 5caC through the BER pathway [16] underwent a slight decline with age in PBMC. TDG is historically known for its anti-mutagenic role in BER, as it removes the thymine moiety from the G:T mismatches, besides being the main DNA glycosylase involved in active DNA demethylation [43]. It is known that impairments of the DNA damage response are implicated in aging process. In fact, genetic defects of genes belonging to the DNA double strand break and nucleotide excision repair systems cause progeroid syndromes, characterized by premature aging phenotypes [44]. Notably, also age-related dysfunctions of BER enzyme expression and/or activity have been found in several tissues but no specific information about TDG is available so far [45].

The decline of *TET1* and *TET3* transcripts with age coincided with the reduction of the global levels of 5hmC. However, only a weak association between *TET1* and 5hmC was evidenced suggesting that the drop of DNA hydroxymethylation with age is most probably due to a combination of multiple events rather than to deficit of a specific TET enzyme. Primarily, as 5hmC formation requires 5mC as initial substrate, which also undergoes reduction with aging, the decrease in 5hmC might reflect defective 5mC levels. Furthermore, 5hmC accumulation also depends on additional iterative oxidation reactions mediated by TET enzymes themselves. Thus, the impairment of TET hydroxylase activity by post-translational modification [46,47], together with the availability of their cofactors, such as ascorbate [48] or 2-oxoglutarate [49], may represent additional mechanisms able to impact 5hmC formation in aging.

The analysis of DNA demethylation intermediates in PBMC evidenced that 5hmC reduction in aged individuals coincided with increased of 5caC levels and almost unaltered content of 5fC. Evidence of 5hmC loss and 5caC accumulation has recently been described in human breast cancers and gliomas [50]. Although the conversion rate of 5hmC into 5fC/5caC is 5-10-fold lower than the one of 5mC into 5hmC [15], a modest impact of 5hmC oxidations could account for its reduction in aging. This is remarkable if one considers that 5fC and 5caC are also stable DNA modifications with their own transcriptional/epigenetic functions [51,52]. The production of 5caC, in presence of reduced *TET1* and *TET3* expression in aging, may depend on *TET2*, whose expression was particularly high compared to the other members of the family and independent of aging. Consistently, several reports demonstrated the ability of TET2 to yield 5caC and to convert 5mC-containing DNA into 5caC also without release of the 5hmC intermediate [53]. On the other hand, the persistence of 5caC in aging could depend on *TDG* down-regulation or on the impairment of the TDG-BER activity. Notably, TET1 interacts with TDG and stabilizes its activity enhancing the TDG-dependent excision of 5caC [54]. Hence the decrease of *TET1* in aging could contribute to 5caC accumulation in addition to 5hmC decline. Moreover, it is to be considered that the decrease of *TET1* and *TET3* gene expression in aging may have additional outcomes besides their contribution to the 5hmC/5fC/5caC levels. In fact, TET enzymes also exhibit non-catalytic functions in transcriptional regulation and DNA repair [42,54,55].

The above-mentioned interplay between *TDG* and *TET1* was supported by the observed highly significant positive correlation of their transcripts. Further, *TET1* strongly correlated with *DNMT1* and *DNMT3B* transcript levels, which have recently been reported to undergo age-related deregulation in PBMC [10]. *TET1* expression was also linked to *PARP1* and *PARP2* gene expression. PARPs are the main enzymes involved in poly(ADP-ribosylation), which has been implicated in aging [56] and in the control of epigenetic modifications of both histones and DNA [57–59]. In addition, *PARP2* and *TDG*, which are both involved in the BER pathway [45], showed an interesting association. These results suggest that genes belonging to DNA methylation/deme-thylation pathways may be co-regulated in immune cells and possibly in aging. Notably, alternative networks of gene correlations have been shown to characterize aging and disease states as well, which may reflect an aberrant function of common transcriptional controllers [60–62].

Age-related alteration of the immune system contributes to increased susceptibility to infections, autoimmune diseases and cancer in the elderly [63]. In this context, the deregulation of *TET* expression and 5hmC formation in PBMC may impair the immune cell functionality in aging. In particular, TET2 is an ascertained gatekeeper of normal hematopoiesis [64] and inflammatory response [42], while the contribution of TET1 in immunity has only recently been reported. TET1 shows a differential expression during T-cell maturation [65] and mediates the transcriptional inhibition of the pro-inflammatory cytokine Interleukin-1β [66]. More importantly, TET1 contributes to the lineage determination of regulatory T-cells, which are necessary for immune homeostasis by suppressing inflammatory responses [67].

In conclusion, this study examined for the first time the age-associated expression of *TET1-3* and *TDG* genes as well as the content of 5hmC and its derivatives in PBMC deriving from the MARK-AGE study, representing a large cross-sectional European population. The enrolment of individuals from eight different European countries with typical climatic and dietary differences has permitted to monitor the influence of diverse environmental variables. However, although the environment represents one of the most significant modifiers of epigenetics [8], it had limited relevance for the parameters investigated, suggesting that the deregulation of 5hmC and *TET* genes is an inherent feature of aging. Furthermore, the age-dependent decline of *TET1*, *TET3* and *TDG* in association with the decrease of 5hmC and the accumulation of 5caC points out an even more intricate epigenetic landscape in PBMC with aging. All these events could have epigenetic and transcriptional consequences that contribute to change DNA methylation patterns and immunological competence in aged individuals.

## MATERIALS AND METHODS

### Study population, recruitment, data and blood collection

PBMC samples used in the present work derived from donors aged 34-74 years that have been recruited in the context of the MARK-AGE project [21,22]. The PBMC isolation procedure, details of the recruitment procedure and of the collection of anthropometric, clinical and demographic data have been published [68,69].

Briefly, PBMCs deriving from donors of eight European countries were isolated from EDTA-whole blood, obtained by phlebotomy after an overnight fasting, by discontinuous density gradient centrifugation in Percoll and subsequently cryopreserved, stored in liquid nitrogen and then shipped to the MARK-AGE Biobank located at the University of Hohenheim, Stuttgart, Germany. From the Biobank, coded samples were subsequently sent to the Sapienza University of Rome on dry ice, where they were stored in liquid nitrogen.

### RNA extraction and cDNA synthesis

Samples were thawed by incubation at 37°C, followed by dropwise addition of RPMI containing 10% FCS to a final dilution of 1:20. Cells were collected by centrifugation and processed for RNA extraction by using RNeasy Mini Kit (Qiagen) according to the manufacturer's instructions and subjected to DNase I digestion using RNase-free DNase (Qiagen). RNA concentration, purity and integrity were evaluated as previously described [70]. Reverse transcription was carried out using the SuperScript VILO cDNA Synthesis Kit (Invitrogen) on equal amounts of total RNA (0.5 μg).

### Real-time quantitative RT-PCR

The expression of *TET1, TET2, TET3* and *TDG* was determined by quantitative PCR using Taqman Gene Expression Assays (Applied Biosystems) following the manufacturer's protocol on the iCycler IQ detection system (Bio-Rad). Each set of primers and probe showed an efficiency of 90–100%. Assays were performed in duplicate with cDNA equivalent to 30 ng of reverse transcribed RNA. Gene expression analysis was performed by the relative calibrator normalized quantification method using the expression level of the *β-glucuronidase* gene (*GUSB*) as reference [70]. An inter-run calibration sample was used in all plates to correct for the technical variance between the different runs and to compare results from different plates. The calibrator consisted of cDNA prepared from HEK293T cells. Taqman Gene Expression Assays IDs for each set of primers and probe were as follows: Hs00286756_m1 (*TET1*); Hs00758658_m1 (*TET2*); Hs00379125_m1 (*TET3*); Hs00702322_s1 (*TDG*); Hs99999908_m1 (*GUSB*).

### Dot blot assay

DNA was extracted from PBMC with DNeasy Blood & Tissue Kit (QIAGEN), samples denatured in 0.4 M NaOH, 10 mM EDTA at 95°C for 10 min, and then neutralized by adding an equal volume of ice-cold 4 M ammonium acetate (pH 7.0). Two-fold dilutions of DNA samples were spotted on a nitrocellulose membrane Hybond-N+ (Amersham Biosciences) in an assembled Bio-Dot apparatus (Bio-Rad). After vacuum-associated blotting procedure, the membrane was washed with 2X SSC buffer, air-dried and blocked with 5% non-fat milk followed by incubation with primary polyclonal anti-5hmC, 5fC, or 5caC antibodies (Active Motif). By the use of HRP-conjugated secondary antibodies, signals were visualized by chemilumine-scence (Amersham ECL Western Blotting detection reagents). A total of 250 ng of DNA was used for 5hmC detection, while 750 ng of DNA for 5fC and 5caC. The same amount of DNA from multiple samples was pooled into different groups of increasing age for the analysis of 5fC and 5caC levels. To correct for technical variance between replicates and to compare results from different experiments, a control DNA deriving from HEK293T cells was used in all assays. To monitor DNA loading, filters were stained with 0.02% methylene blue (MB) in 0.3 M sodium acetate (pH 5.2). Densitometric analysis was performed by Quantity One Software (Bio-Rad Laboratories) according to manufacturer's instructions. To evaluate the specificity of antibodies, 5mC and 5hmC DNA Standard Set (Zymo Research) was used together with DNA derived from HEK293T cells overexpressing the catalytic domain of TET1 (OE TET1).

### EpiTYPER assay for quantitative DNA methylation analysis

The EpiTYPER assay (Sequenom) was used to quantitatively assess the DNA methylation state of *TET1* and *TDG* CGIs and *TET1* 3′-shore. DNA (1 μg) was bisulfite-converted using the EZ-96 DNA Methylation Kit (Zymo Research) with the following modifications: Incubation in CT buffer was performed for 21 cycles of 15 min at 55°C and 30 sec at 95°C and elution of bisulfite-treated DNA was performed in 100 μl of water. PCR was performed on 10 ng of bisulfite-treated DNA using specific primers. The *TET1* CGIs and *TET1* 3′-shore amplicons mapped in chr10:70,320,251-70,320,466 and chr10:70,321,719-70,322,204 (GRCh37/hg19) regions, respectively. *TDG* amplicon mapped in chr12:104,359,220-104,359,701 (GRCh37/hg19) region.

Bisulfite specific primers were the following:

*TDG*_Forward: aggaagagagGGTTGGTAGTATTTAGATAGTGGTTGG*TDG*_Reverse:

cagtaatacgactcactatagggagaaggctAAAACCCAAAATAACACAATAACCTC

*TET1* CGI_Forward:

aggaagagagGGTTTTTAGTTTTAAGTTTGTATTAGTTTT*TET1* CGI_Reverse:

cagtaatacgactcactataGGGAGAAGGCTATCATACAACCCTACCTACCTCTCC

*TET1* 3′shore_Forward:

aggaagagagTTTAAGTTTTTTGATTTTTGGTTTG*TET1* 3′shore_Reverse:

cagtaatacgactcactatagggagaaggctCTCTTAAAATACCTCTTCCCCTCC

### Statistical analysis

Distribution of *TET1, TET2, TET3*, *TDG* and 5hmC variables was investigated Kolmogorov-Smirnov and Shapiro-Wilk normality tests. Identification of potential critical variables that can affect *TET1, TET2, TET3*, *TDG* mRNAs and 5hmC levels in PBMCs was performed by non-parametric tests (Kruskal-Wallis test or Mann-Whitney U test for two group comparisons) as well as by Generalized Linear Models (GLM). Pairwise comparisons (adjusted for multiple comparisons by Dunn's and Bonferroni's methods for the Kruskal-Wallis and the GLM tests, respectively) were used to identify significant differences between percentile groups of each categorized variables.

Influence of age and the relative impact of confounding variables on analyzed parameters, was investigated by linear regression and also by stratified bootstrap sampling (1000 bootstrap samples). GLM were used to investigate the influence of confounding variables (tested as categorized and continuous variables) on age-related changes of analyzed parameters. The Bonferroni adjustment for multiple comparisons was used to identify differences between subgroups.

The identification of the major variables affecting *TET2* mRNA in PBMCs was performed using decision tree analysis with exhaustive “Chi-squared Automatic Interaction Detector” (CHAID) algorithms. To determine the best split at any node, the CHAID algorithm chooses the predictor variable with the smallest adjusted p-value, i.e., the predictor variable that will yield the most significant split. If the smallest (Bonferroni) adjusted p-value for any predictor is greater than some alpha-to-split value, then no further splits are performed, and the respective node is a terminal node. The process repeats recursively until one of the stopping rules is triggered. In growing the tree the following stopping rules were used: minimum terminal parental node size of 20 cases, minimum terminal child node size of 10 cases and alpha = 0.05 for splitting nodes. The convergence criteria for the Exhaustive CHAID were: epsilon = 0.001 and 100 as the maximum number of iterations before stopping the process.

The potential influence of batch effects was investigated by correcting batch effects with the Partek Genomic Suite 6.6 implemented with the ANOVA tool (Partek Incorporated) using log-transformed variables. We considered each real-time PCR plate (containing 8 samples with study groups not evenly distributed across plates) as a batch. We performed adjustments for batches by applying the following strategies: (i) simple removal of batch effect (including PCR plates as batches in the ANOVA tool); (ii) removal of batch effects attempting to retain differences in age and gender (identified as variable of interest in the ANOVA tool). The same analysis was conducted using log-transformed variables of 5hmC. We considered each dot-blot filter (containing 19 samples with study groups not evenly distributed across plates) as a batch.

All statistical analysis (excluding analysis of batch effects) was carried out using SPSS software (SPSS Inc., Chicago, IL; Version 22.0).

## SUPPLEMENTARY MATERIAL FIGURES AND TABLES



## References

[1] Jones PA (2012). Functions of DNA methylation: islands, start sites, gene bodies and beyond. Nat Rev Genet.

[2] Lowe R, Overhoff MG, Ramagopalan SV, Garbe JC, Koh J, Stampfer MR, Beach DH, Rakyan VK, Bishop CL (2015). The senescent methylome and its relationship with cancer, ageing and germline genetic variation in humans. Genome Biol.

[3] Seisenberger S, Peat JR, Hore TA, Santos F, Dean W, Reik W (2013). Reprogramming DNA methylation in the mammalian life cycle: building and breaking epigenetic barriers. Philos Trans R Soc Lond B Biol Sci.

[4] Horvath S (2013). DNA methylation age of human tissues and cell types. Genome Biol.

[5] Bradley-Whitman MA, Lovell MA (2013). Epigenetic changes in the progression of Alzheimer's disease. Mech Ageing Dev.

[6] Teschendorff AE, West J, Beck S (2013). Age-associated epigenetic drift: implications, and a case of epigenetic thrift?. Hum Mol Genet.

[7] Steegenga WT, Boekschoten MV, Lute C, Hooiveld GJ, de Groot PJ, Morris TJ, Teschendorff AE, Butcher LM, Beck S, Müller M (2014). Genome-wide age-related changes in DNA methylation and gene expression in human PBMCs. Age (Dordr).

[8] Zampieri M, Ciccarone F, Calabrese R, Franceschi C, Bürkle A, Caiafa P (2015). Reconfiguration of DNA methylation in aging. Mech Ageing Dev.

[9] Garagnani P, Bacalini MG, Pirazzini C, Gori D, Giuliani C, Mari D, Di Blasio AM, Gentilini D, Vitale G, Collino S, Rezzi S, Castellani G, Capri M (2012). Methylation of ELOVL2 gene as a new epigenetic marker of age. Aging Cell.

[10] Ciccarone F, Malavolta M, Calabrese R, Guastafierro T, Bacalini MG, Reale A, Franceschi C, Capri M, Hervonen A, Hurme M, Grubeck-Loebenstein B, Koller B, Bernhardt J (2016). Age-dependent expression of DNMT1 and DNMT3B in PBMCs from a large European population enrolled in the MARK-AGE study. Aging Cell.

[11] Hahn MA, Szabó PE, Pfeifer GP (2014). 5-Hydroxymethylcytosine: a stable or transient DNA modification?. Genomics.

[12] Tan L, Shi YG (2012). Tet family proteins and 5-hydroxymethylcytosine in development and disease. Development.

[13] Putiri EL, Tiedemann RL, Thompson JJ, Liu C, Ho T, Choi JH, Robertson KD (2014). Distinct and overlapping control of 5-methylcytosine and 5-hydro-xymethylcytosine by the TET proteins in human cancer cells. Genome Biol.

[14] Nestor CE, Ottaviano R, Reddington J, Sproul D, Reinhardt D, Dunican D, Katz E, Dixon JM, Harrison DJ, Meehan RR (2012). Tissue type is a major modifier of the 5-hydroxymethylcytosine content of human genes. Genome Res.

[15] Ito S, Shen L, Dai Q, Wu SC, Collins LB, Swenberg JA, He C, Zhang Y (2011). Tet proteins can convert 5-methylcytosine to 5-formylcytosine and 5-carboxylcytosine. Science.

[16] Shen L, Wu H, Diep D, Yamaguchi S, D'Alessio AC, Fung HL, Zhang K, Zhang Y (2013). Genome-wide analysis reveals TET- and TDG-dependent 5-methylcytosine oxidation dynamics. Cell.

[17] Inoue A, Shen L, Dai Q, He C, Zhang Y (2011). Generation and replication-dependent dilution of 5fC and 5caC during mouse preimplantation development. Cell Res.

[18] Valinluck V, Sowers LC (2007). Endogenous cytosine damage products alter the site selectivity of human DNA maintenance methyltransferase DNMT1. Cancer Res.

[19] Chouliaras L, van den Hove DL, Kenis G, Keitel S, Hof PR, van Os J, Steinbusch HW, Schmitz C, Rutten BP (2012). Age-related increase in levels of 5-hydroxymethylcytosine in mouse hippocampus is prevented by caloric restriction. Curr Alzheimer Res.

[20] Szulwach KE, Li X, Li Y, Song CX, Wu H, Dai Q, Irier H, Upadhyay AK, Gearing M, Levey AI, Vasanthakumar A, Godley LA, Chang Q (2011). 5-hmC-mediated epigenetic dynamics during postnatal neuro-development and aging. Nat Neurosci.

[21] Bürkle A, Moreno-Villanueva M, Bernhard J, Blasco M, Zondag G, Hoeijmakers JH, Toussaint O, Grubeck-Loebenstein B, Mocchegiani E, Collino S, Gonos ES, Sikora E, Gradinaru D (2015). MARK-AGE biomarkers of ageing. Mech Ageing Dev.

[22] Capri M, Moreno-Villanueva M, Cevenini E, Pini E, Scurti M, Borelli V, Palmas MG, Zoli M, Schön C, Siepelmeyer A, Bernhardt J, Fiegl S, Zondag G (2015). MARK-AGE population: from the human model to new insights. Mech Ageing Dev.

[23] Reinius LE, Acevedo N, Joerink M, Pershagen G, Dahlén SE, Greco D, Söderhäll C, Scheynius A, Kere J (2012). Differential DNA methylation in purified human blood cells: implications for cell lineage and studies on disease susceptibility. PLoS One.

[24] Ciccarone F, Valentini E, Bacalini MG, Zampieri M, Calabrese R, Guastafierro T, Mariano G, Reale A, Franceschi C, Caiafa P (2014). Poly(ADP-ribosyl)ation is involved in the epigenetic control of TET1 gene transcription. Oncotarget.

[25] Ichimura N, Shinjo K, An B, Shimizu Y, Yamao K, Ohka F, Katsushima K, Hatanaka A, Tojo M, Yamamoto E, Suzuki H, Ueda M, Kondo Y (2015). Aberrant TET1 Methylation Closely Associated with CpG Island Methylator Phenotype in Colorectal Cancer. Cancer Prev Res (Phila).

[26] Park JL, Kim HJ, Seo EH, Kwon OH, Lim B, Kim M, Kim SY, Song KS, Kang GH, Kim HJ, Choi BY, Kim YS (2015). Decrease of 5hmC in gastric cancers is associated with TET1 silencing due to with DNA methylation and bivalent histone marks at TET1 CpG island 3′-shore. Oncotarget.

[27] Peng B, Hurt EM, Hodge DR, Thomas SB, Farrar WL (2006). DNA hypermethylation and partial gene silencing of human thymine- DNA glycosylase in multiple myeloma cell lines. Epigenetics.

[28] Chou WC, Chou SC, Liu CY, Chen CY, Hou HA, Kuo YY, Lee MC, Ko BS, Tang JL, Yao M, Tsay W, Wu SJ, Huang SY (2011). TET2 mutation is an unfavorable prognostic factor in acute myeloid leukemia patients with intermediate-risk cytogenetics. Blood.

[29] Gaidzik VI, Paschka P, Späth D, Habdank M, Köhne CH, Germing U, von Lilienfeld-Toal M, Held G, Horst HA, Haase D, Bentz M, Götze K, Döhner H (2012). TET2 mutations in acute myeloid leukemia (AML): results from a comprehensive genetic and clinical analysis of the AML study group. J Clin Oncol.

[30] Wang F, Yang Y, Lin X, Wang JQ, Wu YS, Xie W, Wang D, Zhu S, Liao YQ, Sun Q, Yang YG, Luo HR, Guo C (2013). Genome-wide loss of 5-hmC is a novel epigenetic feature of Huntington's disease. Hum Mol Genet.

[31] Calabrese R, Valentini E, Ciccarone F, Guastafierro T, Bacalini MG, Ricigliano VA, Zampieri M, Annibali V, Mechelli R, Franceschi C, Salvetti M, Caiafa P (2014). TET2 gene expression and 5-hydroxymethylcytosine level in multiple sclerosis peripheral blood cells. Biochim Biophys Acta.

[32] Coppieters N, Dieriks BV, Lill C, Faull RL, Curtis MA, Dragunow M (2014). Global changes in DNA methylation and hydroxymethylation in Alzheimer's disease human brain. Neurobiol Aging.

[33] Ding GL, Huang HF (2014). Role for tet in hyperglycemia-induced demethylation: a novel mechanism of diabetic metabolic memory. Diabetes.

[34] Liu R, Jin Y, Tang WH, Qin L, Zhang X, Tellides G, Hwa J, Yu J, Martin KA (2013). Ten-eleven translocation-2 (TET2) is a master regulator of smooth muscle cell plasticity. Circulation.

[35] Bormann F, Rodríguez-Paredes M, Hagemann S, Manchanda H, Kristof B, Gutekunst J, Raddatz G, Haas R, Terstegen L, Wenck H, Kaderali L, Winnefeld M, Lyko F (2016). Reduced DNA methylation patterning and transcriptional connectivity define human skin aging. Aging Cell.

[36] Tammen SA, Dolnikowski GG, Ausman LM, Liu Z, Kim KC, Friso S, Choi SW (2014). Aging alters hepatic DNA hydroxymethylation, as measured by liquid chromatography/mass spectrometry. J Cancer Prev.

[37] Truong TP, Sakata-Yanagimoto M, Yamada M, Nagae G, Enami T, Nakamoto-Matsubara R, Aburatani H, Chiba S (2015). Age-Dependent Decrease of DNA Hydroxymethylation in Human T Cells. J Clin Exp Hematop.

[38] Zhang X, Ulm A, Somineni HK, Oh S, Weirauch MT, Zhang HX, Chen X, Lehn MA, Janssen EM, Ji H (2014). DNA methylation dynamics during ex vivo differentiation and maturation of human dendritic cells. Epigenetics Chromatin.

[39] Bacalini MG, Gentilini D, Boattini A, Giampieri E, Pirazzini C, Giuliani C, Fontanesi E, Scurti M, Remondini D, Capri M, Cocchi G, Ghezzo A, Del Rio A (2015). Identification of a DNA methylation signature in blood cells from persons with Down Syndrome. Aging (Albany NY).

[40] Prati D, Taioli E, Zanella A, Della Torre E, Butelli S, Del Vecchio E, Vianello L, Zanuso F, Mozzi F, Milani S, Conte D, Colombo M, Sirchia G (2002). Updated definitions of healthy ranges for serum alanine aminotransferase levels. Ann Intern Med.

[41] Oh RC, Hustead TR (2011). Causes and evaluation of mildly elevated liver transaminase levels. Am Fam Physician.

[42] Zhang Q, Zhao K, Shen Q, Han Y, Gu Y, Li X, Zhao D, Liu Y, Wang C, Zhang X, Su X, Liu J, Ge W (2015). Tet2 is required to resolve inflammation by recruiting Hdac2 to specifically repress IL-6. Nature.

[43] Dalton SR, Bellacosa A (2012). DNA demethylation by TDG. Epigenomics.

[44] Garinis GA, van der Horst GT, Vijg J, Hoeijmakers JH (2008). DNA damage and ageing: new-age ideas for an age-old problem. Nat Cell Biol.

[45] Xu G, Herzig M, Rotrekl V, Walter CA (2008). Base excision repair, aging and health span. Mech Ageing Dev.

[46] Ciccarone F, Valentini E, Zampieri M, Caiafa P (2015). 5mC-hydroxylase activity is influenced by the PARylation of TET1 enzyme. Oncotarget.

[47] Nakagawa T, Lv L, Nakagawa M, Yu Y, Yu C, D'Alessio AC, Nakayama K, Fan HY, Chen X, Xiong Y (2015). CRL4(VprBP) E3 ligase promotes monoubiquitylation and chromatin binding of TET dioxygenases. Mol Cell.

[48] Minor EA, Court BL, Young JI, Wang G (2013). Ascorbate induces ten-eleven translocation (Tet) methylcytosine dioxygenase-mediated generation of. 5-hydroxymethylcytosine. J Biol Chem..

[49] Xu W, Yang H, Liu Y, Yang Y, Wang P, Kim SH, Ito S, Yang C, Wang P, Xiao MT, Liu LX, Jiang WQ, Liu J (2011). Oncometabolite 2-hydroxyglutarate is a competitive inhibitor of α-ketoglutarate-dependent dioxygenases. Cancer Cell.

[50] Eleftheriou M, Pascual AJ, Wheldon LM, Perry C, Abakir A, Arora A, Johnson AD, Auer DT, Ellis IO, Madhusudan S, Ruzov A (2015). 5-Carboxylcytosine levels are elevated in human breast cancers and gliomas. Clin Epigenetics.

[51] Bachman M, Uribe-Lewis S, Yang X, Burgess HE, Iurlaro M, Reik W, Murrell A, Balasubramanian S (2015). 5-Formylcytosine can be a stable DNA modification in mammals. Nat Chem Biol..

[52] Wang L, Zhou Y, Xu L, Xiao R, Lu X, Chen L, Chong J, Li H, He C, Fu XD, Wang D (2015). Molecular basis for 5-carboxycytosine recognition by RNA polymerase II elongation complex. Nature.

[53] Crawford DJ, Liu MY, Nabel CS, Cao XJ, Garcia BA, Kohli RM (2016). Tet2 Catalyzes Stepwise 5-Methylcytosine Oxidation by an Iterative and de novo Mechanism. J Am Chem Soc..

[54] Weber AR, Krawczyk C, Robertson AB, Kuśnierczyk A, Vågbø CB, Schuermann D, Klungland A, Schär P (2016). Biochemical reconstitution of TET1-TDG-BER-dependent active DNA demethylation reveals a highly coordinated mechanism. Nat Commun..

[55] Williams K, Christensen J, Pedersen MT, Johansen JV, Cloos PA, Rappsilber J, Helin K (2011). TET1 and hydroxymethylcytosine in transcription and DNA methylation fidelity. Nature.

[56] Bürkle A, Brabeck C, Diefenbach J, Beneke S (2005). The emerging role of poly(ADP-ribose) polymerase-1 in longevity. Int J Biochem Cell Biol..

[57] Guastafierro T, Catizone A, Calabrese R, Zampieri M, Martella O, Bacalini MG, Reale A, Di Girolamo M, Miccheli M, Farrar D, Klenova E, Ciccarone F, Caiafa P (2013). ADP-ribose polymer depletion leads to nuclear Ctcf re-localization and chromatin rearrangement(1). Biochem J..

[58] Nalabothula N, Al-jumaily T, Eteleeb AM, Flight RM, Xiaorong S, Moseley H, Rouchka EC, Fondufe-Mittendorf YN Genome-Wide Profiling of PARP1 Reveals an Interplay with Gene Regulatory Regions and DNA Methylation. PLoS One.

[59] Verdone L, La Fortezza M, Ciccarone F, Caiafa P, Zampieri M, Caserta M (2015). Poly(ADP-Ribosyl)ation Affects Histone Acetylation and Transcription. PLoS One.

[60] Kalamohan K, Periasamy J, Bhaskar Rao D, Barnabas GD, Ponnaiyan S, Ganesan K (2014). Transcriptional coexpression network reveals the involvement of varying stem cell features with different dysregulations in different gastric cancer subtypes. Mol Oncol..

[61] Miller JA, Oldham MC, Geschwind DH (2008). A systems level analysis of transcriptional changes in Alzheimer's disease and normal aging. J Neurosci..

[62] Southworth LK, Owen AB, Kim SK (2009). Aging mice show a decreasing correlation of gene expression within genetic modules. PLoS Genet.

[63] Montecino-Rodriguez E, Berent-Maoz B, Dorshkind K (2013). Causes, consequences, and reversal of immune system aging. J Clin Invest.

[64] Ko M, Rao A (2011). TET2: epigenetic safeguard for HSC. Blood.

[65] Berkley AM, Hendricks DW, Simmons KB, Fink PJ (2013). Recent thymic emigrants and mature naive T cells exhibit differential DNA methylation at key cytokine loci. J Immunol.

[66] Neves-Costa A, Moita LF (2013). TET1 is a negative transcriptional regulator of IL-1β in the THP-1 cell line. Mol Immunol.

[67] Yang R, Qu C, Zhou Y, Konkel JE, Shi S, Liu Y, Chen C, Liu S, Liu D, Chen Y, Zandi E, Chen W, Zhou Y, Shi S (2015). Hydrogen Sulfide Promotes Tet1- and Tet2-Mediated Foxp3 Demethylation to Drive Regulatory T Cell Differentiation and Maintain Immune Homeostasis. Immunity.

[68] Moreno-Villanueva M, Capri M, Breusing N, Siepelmeyer A, Sevini F, Ghezzo A, de Craen AJ, Hervonen A, Hurme M, Schön C, Grune T, Franceschi C, Bürkle A (2015). MARK-AGE standard operating procedures (SOPs): A successful effort. Mech Ageing Dev..

[69] Moreno-Villanueva M, Kötter T, Sindlinger T, Baur J, Oehlke S, Bürkle A, Berthold MR (2015). The MARK-AGE phenotypic database: structure and strategy. Mech Ageing Dev..

[70] Zampieri M, Ciccarone F, Guastafierro T, Bacalini MG, Calabrese R, Moreno-Villanueva M, Reale A, Chevanne M, Bürkle A, Caiafa P (2010). Validation of suitable internal control genes for expression studies in aging. Mech Ageing Dev..

