# Supplementary material for: Analysis of the machinery and intermediates of the 5hmC-mediated DNA demethylation pathway in aging on samples from the MARK-AGE Study
Source: Aging (Albany NY). 2016 Aug 29;8(9):1896–915. doi: 10.18632/aging.101022 (PMC5076444; doi:10.18632/aging.101022)
Supplement: Supplementary file 1 [file aging-08-1896-s001.pdf]

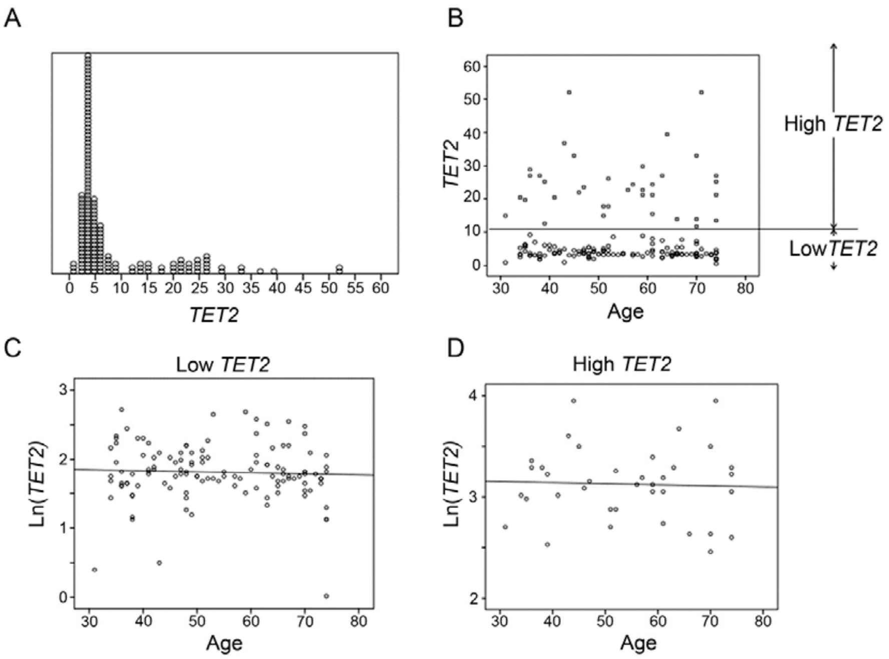

Supplementary Figure 1. Bimodal distribution of *TET2* mRNA levels in PBMC. (**A**)Cluster analysis was performed on *TET2* values data by two step cluster analysis implemented in SPSS package (based on log-likelihood distance measure) using Schwaz's Bayesian as clustering criterion. The tool automatically identifies two clusters separated around the value of 10.0. The cut-off is slightly above the 75th percentile value (8.3). (**B**) *TET2* values showing a spread distribution across age. The subjects assigned to each cluster of *TET2* were named "low *TET2*" and "high *TET2*". The panels below show the linear association with age of Ln(*TET2*) data with "low" (**C**) and "high" (**D**) levels of expression. Linear regression showed no significant changes with age for both classes.

Supplementary Table 1. Regression analysis of *TET1*mRNA levels in PBMC†

Model		Coefficients		Bootstrap for Coefficients		
		B ± SE	Beta	Bias	Sig	95% CI
TET1	(Constant)	0.266 ± 0.024		0.001	0.001	0.227;0.309
	Age (y)	-0.001±0.000	-0.239	< 0.001	0.001	-0.002;-0.001
**Ln(*TET1*)**	(Constant)	-1.316±0.113		0.005	0.001	-1.533;-1.112
	Age (y)	-0.008±0.002	-0.271	< 0.001	0.001	-0.011;-0.004
**Ln(*TET1*) B.C.**	(Constant)	-1.349±0.098		< 0.001	0.001	-1.529;-1.148
	Age (y)	-0.007±0.002	-0.288	< 0.001	0.001	-0.010;-0.004
**Ln(*TET1*) B.C._age;gender**	(Constant)	-1.273±0.097		0.000	0.001	-1.458;-1.089
	Age (y)	-0.008±0.002	-0.341	< 0.001	0.001	-0.012;-0.005

† Regression analysis was performed by using as dependent variable: *TET1* non-transformed data; Ln(*TET1*) data; Ln(*TET1*) data after batch correction (B.C); and Ln(*TET1*) data after batch correction retaining age and gender differences (B.C._age;gender). Bootstrap results are based on 1000 stratified (by recruitment center and gender) bootstrap samples.

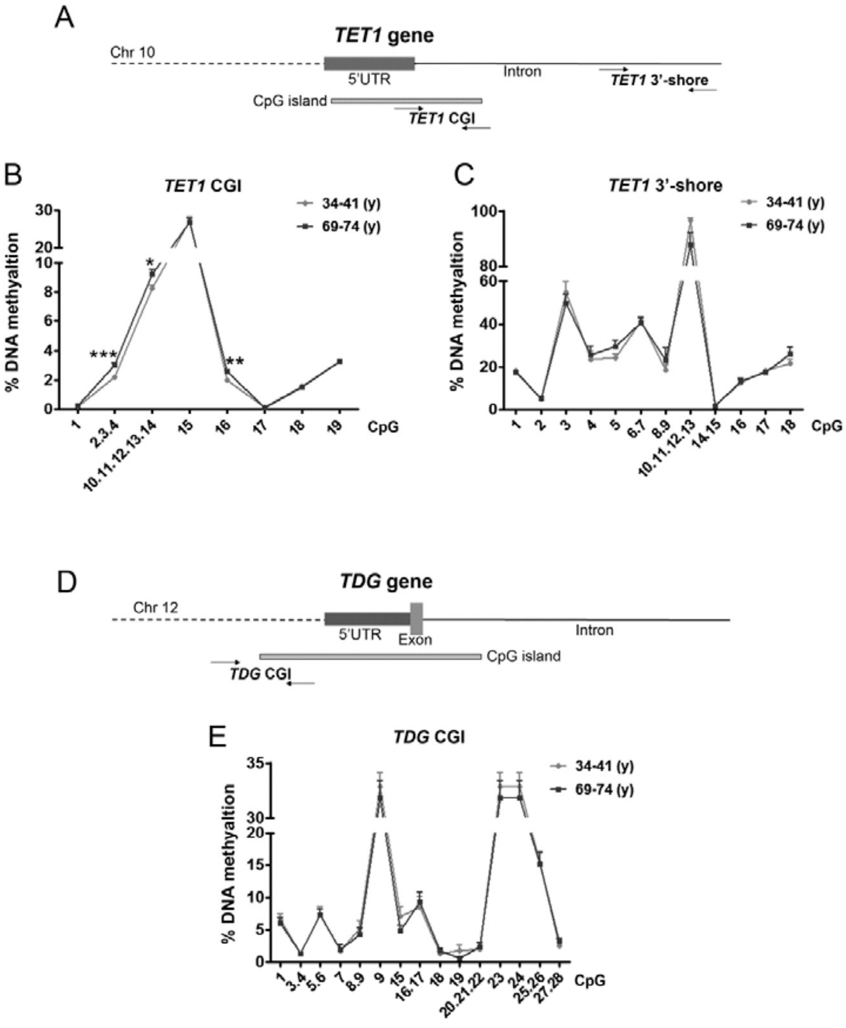

Supplementary Figure 2. DNA methylation analysis of *TET1* and *TDG*regulatory regions. (**A**) Schematic representation of *TET1* gene in which the localization of *TET1* CGI and *TET1* 3'-shore are shown. Age-related changes of DNA methylation in *TET1* CGI (**B**) and *TET1* 3'-shore (**C**) between the groups of young (< 41y) and old (>69 y) individuals are shown. (**D**) Schematic representation of *TDG* gene, in which the localization of CGI is shown. (**E**) Age-related changes of DNA methylation between the groups of young (< 41y) and the old (>69 y) individuals in the *TDG* CGI. Statistical significance was obtained using the Mann-Whitney test (*P < 0.05; **P < 0.01; ***P < 0.001). n(34-41)=36; n(69-74)=34. (y)= years.

.

Supplementary Table 2. Regression analysis of *TET3* mRNA levels in PBMC†

Model		Coefficients		Bootstrap for Coefficients		
		B ± SE	Beta	Bias	Sig	95% CI
TET3	(Constant)	3.684 ± 0.270		0.002	0.001	3.177;4.202
	**Age (y)**	-0.010±0.005	-0.157	< 0.001	0.025	-0.019;-0.001
**Ln(*TET3*)**	(Constant)	1.296±0.086		0.003	0.001	1.136;1.443
	**Age (y)**	-0.004±0.001	-0.172	< 0.001	0.012	-0.006;-0.001
**Ln(*TET3*) B.C.**	(Constant)	1.308±0.070		-0.001	0.001	1.180;1.439
	**Age (y)**	-0.004±0.001	-0.218	< 0.001	0.005	-0.006;-0.001
**Ln(*TET3*) B.C._age;gender**	(Constant)	1.361±0.071		0.002	0.001	1.233;1.493
	**Age (y)**	-0.005±0.001	-0.269	< 0.001	0.001	-0.007;-0.005

† Regression analysis was performed by using as dependent variable: *TET3* non-transformed data; Ln(*TET3*) data; Ln(*TET3*) data after batch correction (B.C); and Ln(*TET3*) data after batch correction retaining age and gender differences (B.C._age;gender). Bootstrap results are based on 1000 stratified (by recruitment center and gender) bootstrap samples.

Supplementary Table 3. Influence of selected factors and covariates on age-related changes of TET1expression†

Test of Model Effects		*TET1*		Ln(*TET1*)		Ln(*TET1*) B.C.		Ln(*TET1*) B.C._age;gender	
**Variables**		Type III		Type III		Type III		Type III	
	**df**	**Wald Chi-Square**	**Sig.**	**Wald Chi-Square**	**Sig.**	**Wald Chi-Square**	**Sig.**	**Wald Chi-Square**	**Sig.**
**(Intercept)**	1	84.628	<0.001	625.562	<0.001	856.957	<0.001	861.589	<0.001
**Center**	7	14.837	0.038	9.575	0.214	7.588	0.370	6.795	0.451
**Gender**	1	0.405	0.525	0.716	0.398	0.970	0.325	1.497	0.221
**Age groups**	2	5.544	0.063	5.884	0.053	6.138	0.046	9.690	0.008
**Lympho/mono**	1	6.152	0.013	9.594	0.002	14.020	<0.001	13.356	<0.001

†Analysis was performed by GLM using linear model with identity link-function considering as dependent variable: *TET1* non-transformed data; Ln(*TET1*) data; Ln(*TET1*) data after batch correction (B.C); and Ln(*TET1*) data after batch correction retaining age and gender differences (B.C._age;gender). Model: (Intercept), center, gender, age groups, lympho/mono (included in the model as continuous variable).

Supplementary Table 4. Regression analysis of TET1 mRNA levels with age considering lympho/mono ratio†

Model		Coefficients B ± SE	Coefficients Beta	Bootstrap for Coefficients Bias	Bootstrap Sig	Bootstrap 95% CI
TET1	(Constant)	0.210		<0.001	0.001	0.154;0.270
	**Age (y)**	-0.001 ± 0.0004	-0.188	<0.001	0.001	-0.002;0.000
	**Lympho/mono**	0.009 ± 0.003	0.193	<0.001	0.005	0.002;0.015
**Ln(*TET1*)**	(Constant)	-1.661 ± 0.152		<0.001	0.001	-1.926;-1.360
	**Age (y)**	-0.006 ± 0.002	-0.203	<0.001	0.004	-0.009;-0.002
	**Lympho/mono**	0.054 ± 0.015	0.241	<0.001	0.003	0.025;0.085
**Ln(*TET1*) B.C.**	(Constant)	-1.673		-0.015	0.001	-1.925;-1.439
	**Age (y)**	-0.005 ± 0.002	-0.224	<0.001	0.001	-0.008;-0.002
	**Lympho/mono**	0.053 ± 0.013	0.275	0.001	0.001	0.031;0.082
**Ln(*TET1*) B.C._age;gender**	(Constant)	-1.590		-0.004	0.001	-1.833;-1.327
	**Age (y)**	-0.007 ± 0.002	-0.279	<0.001	0.001	-0.010;-0.004
	**Lympho/mono**	0.052 ± 0.012	0.267	<0.001	0.001	0.027;0.078

† Regression analysis was performed by using as dependent variable: *TET1* not-transformed data; Ln(*TET1*) data; Ln(*TET1*) data after batch correction (B.C); and Ln(*TET1*) data after batch correction retaining age and gender differences (B.C._age;gender). Bootstrap results are based on 1000 stratified (by recruitment center and gender) bootstrap samples.

Supplementary Table 5. Influence of selected factors and covariates on age-related changes of TET2 expression†

Test of Model Effects		*TET2*		Ln(*TET2*)		Ln(*TET2*) B.C.		Ln(*TET2*) B.C._age;gender	
		Type III		Type III		Type III		Type III	
**Variables**	**df**	**Wald Chi-Square**	**Sig.**	**Wald Chi-Square**	**Sig.**	**Wald Chi-Square**	**Sig.**	**Wald Chi-Square**	**Sig.**
**(Intercept)**	1	28.451	<0.001	118.959	<0.001	132.782	<0.001	132.671	<0.001
**Center**	7	5.158	0.641	3.342	0.852	6.244	0.512	5.507	0.598
**Gender**	1	3.306	0.069	3.933	0.047	2.523	0.112	3.441	0.064
**Age groups**	2	1.898	0.387	4.781	0.092	2.019	0.364	2.245	0.326
**Lympho/mono**	1	2.281	0.131	5.942	0.015	4.789	0.029	4.796	0.029

†Analysis was performed by GLM using linear model with identity link-function considering as dependent variable: *TET2*not-transformed data; Ln(*TET2*) data; Ln(*TET2*) data after batch correction (B.C); and Ln(*TET2*) data after batch correction retaining age and gender differences (B.C._age;gender). Model: (Intercept), center, gender, age groups, lympho/mono (included in the model as continuous variable).

Supplementary Table 6. Influence of selected factors and covariates on age-related changes of *TET3* expression†

Test of Model Effects		*TET3*		Ln(*TET3*)		Ln(*TET3*) B.C.		Ln(*TET3*) B.C._age;gender	
Variables		Type III		Type III		Type III		Type III	
	df	Wald Chi-Square	Sig.	Wald Chi-Square	Sig.	Wald Chi-Square	Sig.	Wald Chi-Square	Sig.
**(Intercept)**	1	322.147	<0.001	416.554	<0.001	592.997	<0.001	624.541	<0.001
**Center**	7	19.893	0.006	17.491	0.014	15.306	0.032	13.952	0.052
**Gender**	1	6.964	0.008	8.302	0.004	4.650	0.031	5.990	0.014
**Age groups**	2	9.621	0.008	9.914	0.007	18.615	<0.001	30.389	<0.001
**Lympho/mono**	1	1.211	0.271	2.918	0.088	3.590	0.058	4.913	0.027

†Analysis was performed by GLM using linear model with identity link-function considering as dependent variable: *TET3* not-transformed data; Ln(*TET3*) data; Ln(*TET3*) data after batch correction (B.C); and Ln(*TET3*) data after batch correction retaining age and gender differences (B.C._age;gender). Model: (Intercept), center, gender, age groups, lympho/mono (included in the model as continuous variable).

Supplementary Table 7. Regression analysis of *TDG* mRNA levels in PBMC†

Model		Coefficients		Bootstrap for Coefficients		
		B ± SE	Beta	Bias	Sig	95% CI
TDG	(Constant)	-0.246 ± 0.026		<0.001	0.001	0.195;0.303
	**Age (y)**	-0.001±0.000	-0.161	<0.001	0.050	-0.002;-3.349E^{-5}
**Ln(*TDG*)**	(Constant)	1.436±0.109		0.003	0.001	-1.631;-1.234
	**Age (y)**	-0.005±0.002	-0.194	<0.001	0.005	-0.009;-0.001
**Ln(*TDG*) B.C.**	(Constant)	-1.519±0.095		-0.001	0.001	-1.721;-1.325
	**Age (y)**	-0.004±0.002	-0.161	<0.001	0.034	-0.007;-1.777E^{-5}
**Ln(*TDG*) B.C._age;gender**	(Constant)	-1.485 ±0.094		0.003	0.001	-1.676;-1.292
	**Age (y)**	-0.004±0.002	-0.188	<0.001	0.020	-0.007;-0.005

† Regression analysis was performed by using as dependent variable: *TDG* not-transformed data; Ln(*TDG*) data; Ln(*TDG*) data after batch correction (B.C); and Ln(*TDG*) data after batch correction retaining age and gender differences (B.C._age;gender). Bootstrap results are based on 1000 stratified (by recruitment center and gender) bootstrap samples.

Supplementary Table 8. Influence of selected factors and covariates on age-related changes of *TDG* expression†

Test of Model Effects		*TDG*		Ln(*TDG*)		Ln(*TDG*) B.C.		Ln(*TDG*) B.C._age;gender	
Variables		Type III		Type III		Type III		Type III	
	df	Wald Chi-Square	Sig.	Wald Chi-Square	Sig.	Wald Chi-Square	Sig.	Wald Chi-Square	Sig.
**(Intercept)**	1	111.058	<0.001	542.769	<0.001	773.422	<0.001	764.387	<0.001
**Center**	7	40.802	<0.001	36.851	<0.001	32.153	<0.001	29.293	<0.001
**Gender**	1	1.082	0.297	0.821	0.365	0.931	0.334	1.129	0.288
**Age groups**	2	2.079	0.354	3.411	0.182	3.167	0.205	4.246	0.120
**Lympho/mono**	1	0.524	0.469	0.000	0.992	1.026	0.311	0.880	0.348

†Analysis was performed by GLM using linear model with identity link-function considering as dependent variable: *TDG* not-transformed data; Ln(*TDG*) data; Ln(*TDG*) data after batch correction (B.C); and Ln(*TDG*) data after batch correction retaining age and gender differences (B.C._age;gender). Model: (Intercept), center, gender, age groups, lympho/mono (included in the model as continuous variable).

Supplementary Table 9. Regression analysis of 5hmClevels in PBMC†

Model		Coefficients		Bootstrap for Coefficients		
		B ± SE	Beta	Bias	Sig	95% CI
**5hmC**	(Constant)	1.341 ± 0.106		0.001	0.001	1.165;1.527
	**Age (y)**	-0.004±0.002	-0.190	<0.001	0.008	-0.008;-0.001
**Ln(5hmC)**	(Constant)	0.298±0.102		-0.006	0.001	0.131;0.462
	**Age (y)**	-0.004±0.002	-0.199	<0.001	0.005	-0.007;-0.001
**Ln(5hmC) B.C.**	(Constant)	0.303±0.092		0.002	0.001	0.145;0.462
	**Age (y)**	-0.005±0.002	-0.227	<0.001	0.004	-0.007;-0.002
**Ln(5hmC) B.C._age;gender**	(Constant)	0.353±0.092		0.001	0.001	0.205;0.503
	**Age (y)**	-0.005±0.002	-0.266	<0.001	0.002	-0.008;-0.003

† Regression analysis was performed by using as dependent variable: 5hmC not-transformed data; Ln(5hmC) data; Ln(5hmC) data after batch correction (B.C); and Ln(5hmC) data after batch correction retaining age and gender differences (B.C._age;gender). Bootstrap results are based on 1000 stratified (by recruitment center and gender) bootstrap samples.

Supplementary Table 10. Influence of selected factors and covariates on age-related changes of 5hmC levels†

Test of Model Effects		5hmC		Ln(5hmC)		Ln(5hmC) B.C.		Ln(5hmC) B.C._age;gender	
		Type III		Type III		Type III		Type III	
**Variables**	**df**	**Wald Chi-Square**	**Sig.**	**Wald Chi-Square**	**Sig.**	**Wald Chi-Square**	**Sig.**	**Wald Chi-Square**	**Sig.**
**(Intercept)**	1	220.480	<0.001	0.135	0.713	0.348	0.555	0.369	0.543
**Center**	7	13.213	0.067	15.579	0.029	16.095	0.024	15.522	0.030
**Gender**	1	0.916	0.339	1.755	0.185	3.273	0.070	4.089	0.043
**Age groups**	2	10.599	0.005	13.316	0.001	12.444	0.002	14.633	0.001
**Lympho/mono**	1	0.014	0.906	0.113	0.737	0.010	0.919	0.033	0.857

†Analysis was performed by GLM using linear model with identity link-function considering as dependent variable: 5hmCnot-transformed data; Ln(5hmC) data; Ln(5hmC) data after batch correction (B.C); and Ln(5hmC) data after batch correction retaining age and gender differences (B.C._age;gender). Model: (Intercept), center, gender, age groups, lympho/mono (included in the model as continuous variable).
